# An Inconsistent Canadian Provincial and Territorial Response During the Early COVID-19 Pandemic

**DOI:** 10.3389/fpubh.2021.708903

**Published:** 2021-09-27

**Authors:** Amelie Cyr, Prosanta Mondal, Gregory Hansen

**Affiliations:** ^1^Department of Pediatrics, College of Medicine, University of Saskatchewan, Saskatoon, SK, Canada; ^2^Clinical Research Support Unit, College of Medicine, University of Saskatchewan, Saskatoon, SK, Canada; ^3^Division of Pediatric Critical Care, Jim Pattison Children's Hospital, Saskatoon, SK, Canada

**Keywords:** Canada, COVID-19 pandemic, disease outbreak, population health, public health

## Abstract

**Objectives:** According to the World Health Organization (WHO), an early and consistent international and national response is needed to control a pandemic's spread. In this analysis, we evaluate the coordination of Canada's early response to the coronavirus (COVID-19) pandemic in terms of public health interventions and policies implemented in each province and territory.

**Methods:** Retrospective data was obtained from publicly accessible websites maintained by federal, provincial and territorial governmental agencies. Consistent with WHO's spreading of the disease pandemic action, individual and community-based public health interventions and policies were the focus. Time of intervention or policy, and COVID-19 cases per million at time of intervention was recorded for each province and territory.

**Results:** Most public health interventions and policies demonstrated wide time ranges of implementation across individual provinces and territories. At time of implementation, there were also wide variations in the number of positive COVID-19 cases in these jurisdictions. Cases per million per implemented day were also not similar across interventions or policy, suggesting that other factors may have been preferentially considered.

**Conclusions:** Whether an earlier and more structured national approach would have lessened the pandemic's burden is uncertain, calls for greater federal coordination and leadership should to examined.

## Introduction

The World Health Organization (WHO) was first made aware of a “viral pneumonia” occurring in Wuhan, China, on December 31st, 2019. Rapidly, the novel coronavirus spread outside of the Chinese borders, and by January 30th, it received the WHO's highest level of alarm (1). By March 2020, COronaVIrus Disease 2019 (COVID-19) was declared a global pandemic, and by April 2020, there were over one million cases confirmed across the world (1).

The need for an early and structured public health intervention plan to limit COVID-19's progression became paramount. In retrospect, the resounding cornerstone of the WHO's pandemic preparedness and response became leadership and coordinated actions at the global and national level (2). In Canada, its federal response initially focused on screening and isolating international travelers at risk (3). However, as the number of cases throughout the country increased, the federal and provincial responses shifted toward a more complete public health approach. The federal government expanded its actions to travel restrictions, control of borders as well as issuing public health guidance (4) while the provincial responses were focused on case management and isolation, limitation of gatherings, health workforce reassignment and closure of certain communities and non-essential businesses.

Some concerns were raised after the first wave of the COVID-19 pandemic in regards to differences between provincial interventions. In Canada, the public healthcare system is largely decentralized, as the primary responsibilities are shouldered provincially and territorially (5). However, the “Federal/Provincial/Territorial (F/P/T) Public Health Response Plan for Biological Events,” outlines that “the key objective of this plan is improving effective engagement amongst public health, health care delivery and health emergency management authorities during a coordinated F/P/T response” (6).

In our perspective, we reviewed Canada's early response to the COVID-19 pandemic in terms of public health policies and interventions. More specifically, we examined how implementation differed across provinces and if Canada met its objective of having an “overarching, consistent and coordinated plan” across the country (6).

## Materials and Methods

### Definition

Canada's early response was defined from the first case reported on January 25th, 2020 (7) to the end of April 2020. The 3-month time interval captured the most restrictive public health policy measures and pandemic interventions for the majority of provinces and territories, just before the spring lockdown was gradually reopened.

### Variables

Time of intervention or policy, and COVID-19 cases per million at time of policy/intervention was recorded for each province and territory. Day #0 for each policy/intervention was considered when the first province implemented it. Consistent with WHO's reducing the spread of disease preparedness (2) during a pandemic, and individual and community-based public health interventions and policies were the focus. Mortality data was not captured, as death rates remained very low during the early response.

Data on daily numbers of COVID-19 cases for each province were collected from the Government of Canada's public access epidemiological tool (7). We collected data from January 25th 2020, which is when the first Canadian COVID-19 case was reported, to the end of April 2020, which is when most provinces adopted their early pandemic response interventions. If some provinces adopted their interventions later than April 2020, we expanded our data collection to those specific dates.

Data for public health interventions and policies as well as their date of implementation were extracted from provincial or territorial websites, which included the Governments of British Columbia (8), Alberta (9), Saskatchewan (10), Manitoba (11), Ontario (12), Quebec (13), New Brunswick (14), Nova Scotia (15), Prince Edward Island (16), Newfoundland and Labrador (17), Yukon (18), Northwest Territories (19), and Nunavut (20).

### Statistical Analysis

Continuous variables were summarized as means and standard deviations. We used Pearson's correlation to measure the correlation between the days of intervention and the number of COVID-19 cases. The days of intervention and the number of COVID-19 cases were created by taking the averages of corresponding values from the provinces. Statistical analyses were done using SAS 9.4 (SAS Institute Inc., Cary, NC, USA).

## Results

**Figure 1:**
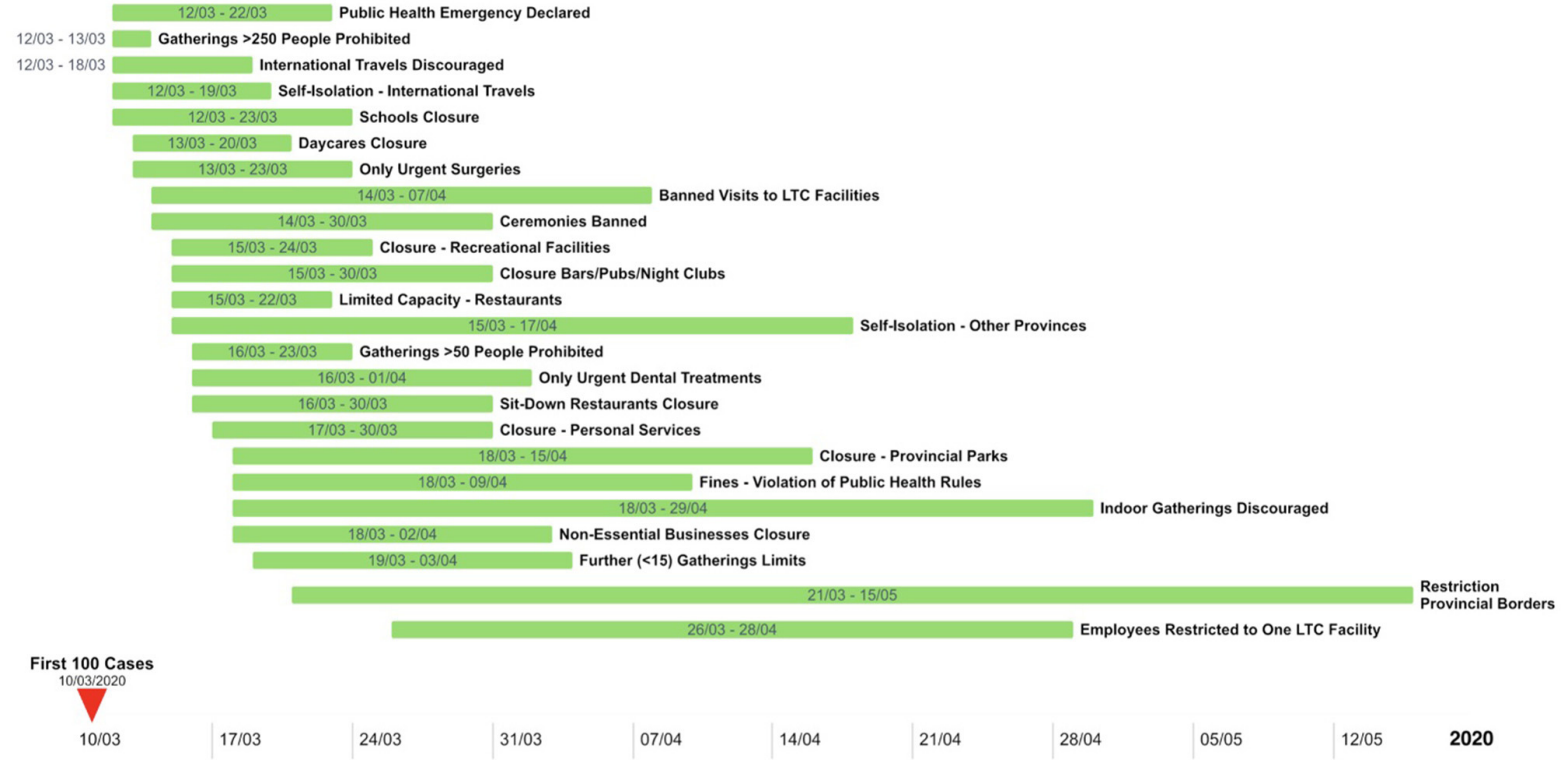
Timeline range of provincial and territorial public health interventions and policies.

The provincial and territorial public health interventions are summarized in Table 1 and Figure 1 by date of implementation. Thirteen public health interventions (57%) out of the 24 analyzed were implemented by all provinces and territories across Canada. Most public health interventions showed wide implementation date ranges across provinces with large standard deviations. For example, declaring public health emergencies was adopted by all jurisdiction within a 10 day period, and had a standard deviation of 2.30 days. Restricting provincial border restrictions however, was only adopted by 46% (*n* = 6) provinces and/or territories, and had an implementation date range of 55 days and a standard deviation of 22 days.

**Table 1:** Public health interventions during the early COVID-19 pandemic analyzed by date of implementation.

**Intervention or policy**	**First province/territory implementing policy**	**Last province/territory implementing policy**	**Date range between first and last province/territory (days)**	**Mean (days)**	**Standard deviation (days)**	**Number of provinces/territories implementing policy (out of 13)**
Public health emergency declared	Quebec (March 12th)	Nova Scotia (March 22nd)	10	5.85	2.30	13
International travels discouraged	B.C., QC, N.L. (March 12th)	New Brunswick (March 18th)	6	2.00	1.87	13
Gatherings >250 people prohibited	B.C., N.B. (March 12th)	AB, MB, SK, ON, QC, NS (March 13th)	1	0.75	0.46	8
Gatherings >50 people prohibited	BC, ON, QC, PEI, YT (March 16th)	Nunavut (March 23rd)	7	1.33	2.02	12
Further (<15) gathering limits	New Brunswick (March 19th)	P.E.I. (April 3rd)	15	5.92	4.17	12
Indoor gatherings discouraged	QC, N.B. (March 18th)	Nunavut (April 29th)	42	12.00	11.26	11
Self-isolation – international travel	BC, AB, QC (March 12th)	New Brunswick (March 19th)	7	2.31	1.93	13
Self-isolation – from other province	Nunavut (March 15th)	Manitoba (April 17th)	33	9.38	9.97	8
Restriction provincial borders	PEI, NT (March 21st)	NL (May 15th)	55	10.33	21.93	6
School closure	Ontario (March 12th)	MB, NS, PEI (March 23rd)	11	5.08	3.80	13
Closure daycares	Quebec (March 13th)	Manitoba (March 20th)	7	3.33	1.87	9
Closure bars, pubs and night clubs	Quebec (March 15th)	Manitoba (March 30th)	15	4.46	4.12	13
Limited capacity – restaurants	Quebec (March 15th)	Yukon (March 22nd)	7	3.00	2.27	8
Sit-down restaurants closure	Ontario (March 16th)	Manitoba (March 30th)	14	5.33	4.01	12
Non-essential business closure	P.E.I, NU (March 18th)	Yukon (April 2nd)	15	6.00	4.88	13
Closure – personal services	P.E.I. (March 17th)	Manitoba (March 30th)	13	4.92	3.59	13
Closure recreational facilities	Quebec (March 15th)	British Columbia (March 24th)	9	3.69	2.95	13
Ceremonies banned	P.E.I. (March 14th)	Manitoba (March 30th)	16	6.54	4.67	13
Closure provincial parks	Ontario (March 18th)	Quebec (April 15th)	28	9.90	10.18	10
Banned visits to LTC facilities	ON, QC (March 14th)	Alberta (April 7th)	24	6.38	7.24	13
Employees restricted to one LTC facility	British Columbia (March 26th)	Newfoundland (April 28th)	33	20.50	12.21	6
Fines – violation public health rules	Newfoundland (March 18th)	Manitoba (April 9th)	22	8.00	6.15	13
Only urgent dental treatments	ON, QC (March 16th)	MB, NU (April 1st)	16	5.54	5.56	13
Only urgent surgeries	Quebec (March 13th)	SK, MB (March 23rd)	10	4.85	2.88	13

Public health interventions based on the number of cases per millions are summarized in Table 2. For most interventions, there were wide standard deviations demonstrating heterogeneity in the number of cases when provinces or territories adopted each public health intervention. Restricting gatherings of >250 people was implemented with 3.88 mean provincial cases per million, whereas restricting employees to one long term facility was implemented with 335.85 mean provincial cases per million.

**Table 2:** Public health interventions during the early COVID-19 pandemic analyzed by cases per province or territory at the time of implementation.

**Intervention or policy**	**First province/territory implementing policy**	**Last province/territory implementing policy**	**Mean (cases/1M)**	**Standard deviation (cases/1M)**
Public health emergency declared	Quebec (2 cases/1M)	Nova Scotia (29 cases/1M)	12.85	13.53
Non-essential international travels discouraged	BC (10 cases/1M) QC (2 cases/1M) NL (0 case/1M)	New Brunswick (15 cases/1M)	4.31	4.75
Gatherings >250 people prohibited	BC (10 cases/1M) N.B. (1 case/1M)	AB (8 cases/1M) MB (3 cases/1M) SK (2 cases/1M) ON (5 cases/1M) QC (2 cases/1M) NS (0 case/1M)	3.88	3.52
Gatherings >50 people prohibited	BC (20 cases/1M) ON (12 cases/1M) QC, PEI (6 cases/1M) YT (0 case/1M)	Nunavut (0 case/1M)	9.08	7.98
Further (<15) gathering restrictions	New Brunswick (15 cases/1M)	P.E.I. (140 cases/1M)	60.33	46.88
Indoor gatherings discouraged	QC (11 cases/1M) N.B. (15 cases/1M)	Nunavut (0 case/1M)	94.09	67.45
Self-isolation international travelers	BC (10 cases/1M) AB (6 cases/1M) QC (2 cases/1M)	New Brunswick (15 cases/1M)	4.23	4.51
Self-isolation – returning from other province	Nunavut (0 case/1M)	Manitoba (187 cases/1M)	45.50	60.19
Restricted provincial borders	PEI (13 cases/1M) NT (22 cases/1M)	NL (498 cases/1M)	99.50	195.61
School closure	Ontario (4 cases/1M)	MB (15 cases/1M) NS (42 cases/1M) PEI (19 cases/1M)	10.92	14.22
Closure daycares	Quebec (2 cases/1M)	Manitoba (12 cases/1M)	9.00	5.22
Closure bars, pubs, clubs	Quebec (4 cases/1M)	Manitoba (96 cases/1M)	23.77	26.68
Limited capacity – restaurants	Quebec (4 cases/1M)	Yukon (57 cases/1M)	20.75	18.19
Sit-down restaurants closed	Ontario (12 cases/1M)	Manitoba (96 cases/1M)	42.08	34.23
Non-essential business closure	P.E.I (6 cases/1M) NU (0 case/1M)	Yukon (171 cases/1M)	71.54	61.81
Closure - personal services	P.E.I. (6 cases/1M)	Manitoba (96 cases/1M)	46.38	40.72
Closure recreational facilities	Quebec (4 cases/1M)	British Columbia (122 cases/1M)	22.08	33.41
Ceremonies banned	P.E.I. (6 cases/1M)	Manitoba (96 cases/1M)	34.15	39.16
Closure provincial parks	Ontario (15 cases/1M)	Quebec (1751 cases/1M)	245.10	536.39
Banned visits to LTC facilities	ON (7 cases/1M) QC (2 cases/1M)	Alberta (309 cases/1M)	39.54	85.07
Employees restricted to one LTC facility	British Columbia (143 cases/1M)	Newfoundland (494 cases/1M)	335.83	170.97
Fines - violation public health rules	Newfoundland (6 cases/1M)	Manitoba (175 cases/1M)	68.77	63.70
Only urgent dental treatments	ON (12 cases/1M) QC (6 cases/1M)	MB (132 cases/1M) NU (0 case/1M)	38.46	45.87
Only urgent surgeries	Quebec (2 cases/1M)	SK (56 cases/1M) MB (15 cases/1M)	12.62	16.00

**Figure 2:**
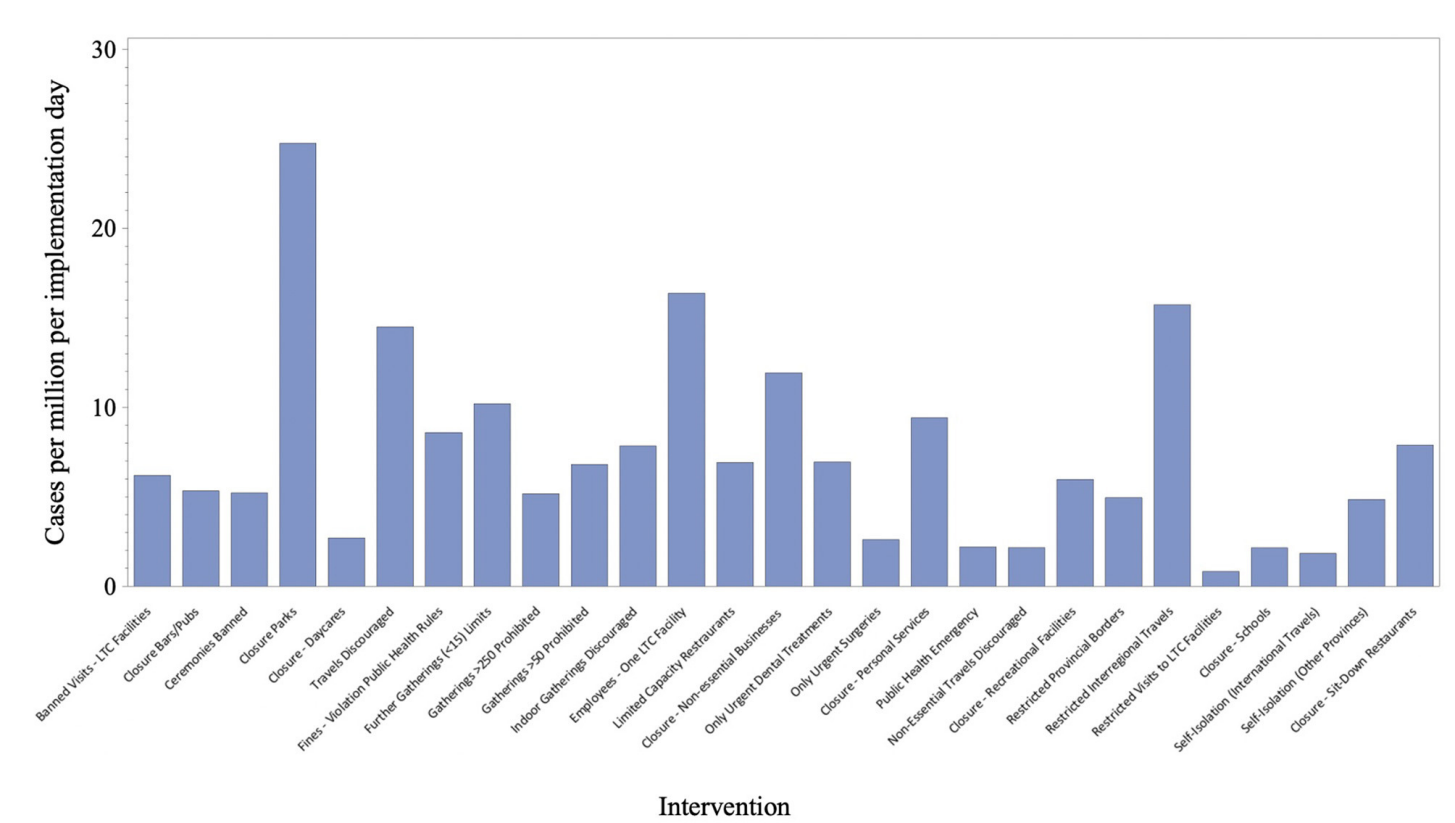
Average cases per million per implementation day.

The correlation between mean days of intervention and the number of COVID-19 cases was positive and significant (rho = 0.89; *p* < 0.0001). However, cases per million per implemented day was not similar across intervention or policy. For example, closure of parks, restricting long term care facility employees and restricted travel had much higher cases than other policies (Figure 2).

## Discussion

Provincial and territorial public health interventions adopted during the early COVID-19 pandemic response showed a large amount of heterogeneity in terms of their implementation timeline across Canada. In fact, 43% of the interventions were not adopted by all. Furthermore, their implementation did not appear to be consistent with the number of positive COVID-19 cases, suggesting other factors may have been considered.

Earlier interventions such as the declaration of a public health emergency and discouraging international travel were widely adopted by all provinces and territories and within a short period of time. However, considering the effectiveness of physical distancing measures to reduce overall community transmission (3, 21) only eight provinces adopted policies limiting gatherings to <250 people. Other establishment closures such as bars, restaurants, non-essential businesses and personal services also demonstrated large variations, despite the utility of physical distancing measures at “mixed-aged venues” (3). Surprisingly, interventions for long term care facilities showed the highest variability, despite the impact of direct measures targeted at reducing COVID-19 transmission to older citizens (3, 22). Only six provinces or territories restricted employees to work in different long-term care facilities in order to limit the spread between them. British Columbia's early approach was the most stringent, and banned visits to long-term care facilities, restricted movements of employees and made masks obligatory (23). Together, the data suggests that provincial and territorial public health policies were not implemented in a coordinated and dependent fashion.

An uncoordinated national approach is important to consider, as half of the provinces/territories did not restrict their borders to domestic travelers including between the most affected and highly populated regions. Newfoundland and Labrador closed their provincial borders early, and through modeling demonstrated how easy an infected traveler can be the cause of new exponential outbreak by solely relaxing travel bans (24). In contrast, the first human-to-human COVID-19 transmission was confirmed in Wuhan, China, on January 20th 2020 (25). Three days later, major non-pharmaceutical interventions were adopted across the country including intercity travel restrictions (26). Based on an epidemiological model, the travel restrictions did “prevent cases being exported from Wuhan to a wider area” (26) and had they been implemented earlier, the outbreak would have been controlled even further. Although a discussion of Wuhan's response vs. Western liberties is beyond the scope of this study, it nevertheless demonstrates the effectiveness of an attentive and coordinated border strategy.

Decisions to implement provincial and territorial public health interventions did not appear to be based on the number of their COVID-19 cases. Escalation of responses that included closure of schools, non-essential businesses and services occurred over a wide range of provincial/territorial positive cases and calendar days. When we considered cases per million per implemented day closure of parks, restricting long term care facility employees and restricting travel were far higher than other policies, suggesting they should have been implemented sooner. In contrast, territories like Nunavut adopted the majority of public health interventions implemented by other provinces with escalating cases, despite not having a positive case documented. Other countries were also reactive during the early COVID-19 pandemic, but implemented national public health interventions uniformly as initial cases were detected within their borders (27). Some jurisdictions were more proactive. Hong Kong, for example, restricted travelers coming from Wuhan, screened returning travelers, and limited travel even before their first case was confirmed (28). With those proactive interventions, Hong Kong was able to contain COVID-19 spread despite its proximity with Wuhan, while avoiding more extreme interventions like complete lockdown (28).

Our findings suggest that Canada's F/P/T Public Health Response Plan for Biological Events did not result in a coordinated and consistent national response.

In March, 2020, reporters commented that Canada did not learn from key lessons after SARS, with its patchwork system of basic information and sparse details on coronavirus communications (29). During the second wave, some provincial leaders were asking for a “pan-Canadian approach to travel” (30) while infectious disease specialists were calling for an “aggressive national strategy” (31). While the consequences of Canada's decentralized approach in regards to morbidity, mortality and economic loss remains uncertain, the degree of scrutiny from well-intentioned medical experts and politicians should compel a thorough review of the Response Plan and the degree of Federal coordination and involvement.

There are at least two limitations that should be addressed. First, public health decisions are based on complicated processes that must consider a plethora of information, factors, and opinions. To suggest otherwise was not our intension, but rather to examine how coordinated the decisions were. The second limitation is more philosophic in nature, as the noted decentralized approach has been suggested to enable provinces and territories to innovate and mobilize quickly. However, elsewhere we have shown the opposite (27) and that the decentralized uncoordinated approach has actually resulted in suboptimal testing, mobilization of the health care system and disease containment.

The World Health Organization has advocated for early coordinated responses during pandemics. In Canada, many provincial and territorial public health interventions were implemented across wide time ranges. Cases per million per implemented day were also not similar, suggesting that other factors may have been preferentially considered for interventions or policies. Whether an earlier and more structured national approach would have lessened the pandemic's burden is uncertain, calls for greater federal coordination and leadership should to examined.

## Data Availability Statement

The original contributions presented in the study are included in the article/supplementary material, further inquiries can be directed to the corresponding author/s.

## Author Contributions

AC and GH developed the central idea of the study. PM provided statistical support. All authors wrote, revised, and approved the final manuscript as submitted.

## Conflict of Interest

The authors declare that the research was conducted in the absence of any commercial or financial relationships that could be construed as a potential conflict of interest.

## Publisher's Note

All claims expressed in this article are solely those of the authors and do not necessarily represent those of their affiliated organizations, or those of the publisher, the editors and the reviewers. Any product that may be evaluated in this article, or claim that may be made by its manufacturer, is not guaranteed or endorsed by the publisher.
